# Traumatic Brain Injury as an Independent Predictor of Futility in the Early Resuscitation of Patients in Hemorrhagic Shock

**DOI:** 10.3390/jcm13133915

**Published:** 2024-07-03

**Authors:** Mahmoud D. Al-Fadhl, Marie Nour Karam, Jenny Chen, Sufyan K. Zackariya, Morgan C. Lain, John R. Bales, Alexis B. Higgins, Jordan T. Laing, Hannah S. Wang, Madeline G. Andrews, Anthony V. Thomas, Leah Smith, Mark D. Fox, Saniya K. Zackariya, Samuel J. Thomas, Anna M. Tincher, Hamid D. Al-Fadhl, May Weston, Phillip L. Marsh, Hassaan A. Khan, Emmanuel J. Thomas, Joseph B. Miller, Jason A. Bailey, Justin J. Koenig, Dan A. Waxman, Daniel Srikureja, Daniel H. Fulkerson, Sarah Fox, Greg Bingaman, Donald F. Zimmer, Mark A. Thompson, Connor M. Bunch, Mark M. Walsh

**Affiliations:** 1 Department of Medical Education, South Bend Campus, Indiana University School of Medicine, South Bend, IN 46617, USA; markaram@iu.edu (M.N.K.); jc111@iu.edu (J.C.); szackari@iu.edu (S.K.Z.); molain@iu.edu (M.C.L.); jorbales@iu.edu (J.R.B.); alebhigg@iu.edu (A.B.H.); jordlain@iu.edu (J.T.L.); hw16@iu.edu (H.S.W.); madandre@iu.edu (M.G.A.); anvthoma@iu.edu (A.V.T.); smitleah@iu.edu (L.S.); markfox@iu.edu (M.D.F.); anntinch@iu.edu (A.M.T.); halfadhl@iu.edu (H.D.A.-F.); 2 Department of Internal Medicine, Saint Joseph Regional Medical Center, Mishawaka, IN 46545, USA; szackariya15@gmail.com (S.K.Z.); samueljthomas1@gmail.com (S.J.T.); mayeweston@gmail.com (M.W.); plmarsh1212@gmail.com (P.L.M.); hassaankhan149@gmail.com (H.A.K.); emmanueljthomas4@gmail.com (E.J.T.); 3 Department of Emergency Medicine, Henry Ford Hospital, Detroit, MI 48202, USA; jmiller6@hfhs.org (J.B.M.); cbunch1@hfhs.org (C.M.B.); 4 Department of Emergency Medicine, Elkhart General Hospital, Elkhart, IN 46515, USA; jbailey@beaconhealthsystem.org; 5 Department of Trauma & Surgical Services, Memorial Hospital, South Bend, IN 46601, USA; jkoenig@beaconhealthsystem.org (J.J.K.); dhfulkerson@beaconhealthsystem.org (D.H.F.); ssfox@beaconhealthsystem.org (S.F.); gbingaman@beaconhealthsystem.org (G.B.); 6 Department of Pathology and Laboratory Medicine, Indiana University School of Medicine, Indianapolis, IN 46601, USA; dwaxman@versiti.org; 7 Versiti Blood Center of Indiana, Indianapolis, IN 46208, USA; 8 Department of Surgery, Memorial Hospital, South Bend, IN 46601, USA; dsrikureja@beaconhealthsystem.org (D.S.); mthompson2@beaconhealthsystem.org (M.A.T.); 9 Department of Neurosurgery, Memorial Hospital, South Bend, IN 46601, USA; 10 Department of Emergency Medicine, Memorial Hospital, South Bend, IN 46601, USA; dzimmer@beaconhealthsystem.org

**Keywords:** traumatic brain injury, futility, resuscitation, massive transfusion, hemorrhage, shock, trauma, emergency

## Abstract

This review explores the concept of futility timeouts and the use of traumatic brain injury (TBI) as an independent predictor of the futility of resuscitation efforts in severely bleeding trauma patients. The national blood supply shortage has been exacerbated by the lingering influence of the COVID-19 pandemic on the number of blood donors available, as well as by the adoption of balanced hemostatic resuscitation protocols (such as the increasing use of 1:1:1 packed red blood cells, plasma, and platelets) with and without early whole blood resuscitation. This has underscored the urgent need for reliable predictors of futile resuscitation (FR). As a result, clinical, radiologic, and laboratory bedside markers have emerged which can accurately predict FR in patients with severe trauma-induced hemorrhage, such as the Suspension of Transfusion and Other Procedures (STOP) criteria. However, the STOP criteria do not include markers for TBI severity or transfusion cut points despite these patients requiring large quantities of blood components in the STOP criteria validation cohort. Yet, guidelines for neuroprognosticating patients with TBI can require up to 72 h, which makes them less useful in the minutes and hours following initial presentation. We examine the impact of TBI on bleeding trauma patients, with a focus on those with coagulopathies associated with TBI. This review categorizes TBI into isolated TBI (iTBI), hemorrhagic isolated TBI (hiTBI), and polytraumatic TBI (ptTBI). Through an analysis of bedside parameters (such as the proposed STOP criteria), coagulation assays, markers for TBI severity, and transfusion cut points as markers of futilty, we suggest amendments to current guidelines and the development of more precise algorithms that incorporate prognostic indicators of severe TBI as an independent parameter for the early prediction of FR so as to optimize blood product allocation.

## 1. Introduction: The Importance of Defining Futility in the Current Blood Product Shortage

Recent shortages of blood products have caused traumatologists to search for reliable predictors of futile resuscitation (FR) in patients with hemorrhagic shock who do not respond to the initial attempts to provide adequate hemostatic resuscitation. The disruption in the delivery and production of blood products caused by the COVID-19 pandemic, as well as the reduction in the number of young people volunteering for blood transfusion, has caused a significant shortage of blood components available for trauma resuscitation. Combined with this is the recent adoption of the 1:1:1 ratio of packed red blood cells (PRBC) to plasma to platelets (PLT) and the increasing use of whole blood hemostatic resuscitation for these patients which can intermittently drain blood banks of much of their blood supply during peak blood component use, even at large trauma centers [1,2,3,4,5,6,7]. The concept of reducing blood product wastage is important in settings of clinical futility to minimize blood product waste for patients with poor prognoses. This situation has forced traumatologists and their blood-banking colleagues to confront the ethical ambiguities inherent in these difficult decisions with specific algorithms that define futility [2,6]. An example is an algorithm which includes the patient’s Sequential Organ Failure Assessment (SOFA) score, the need for ongoing transfusion support, and the likelihood of hemorrhagic arrest [2]. This algorithm is triggered at periodic intervals during the administration of blood products in patients with severe hemorrhage where there is a concern for the futility of continued treatment. This periodic assessment is a precursor to the recent proposition of futility timeouts (FTOs)/transfusion timeouts (TTOs) during the administration of massive transfusion (MT) to severely bleeding trauma patients [2,6,7,8].

As a result, there has been a significant attempt to define specific clinical laboratory and radiologic parameters that will assist in defining FR. Among those clinical parameters are transfusion cut points per hour (and over a specific period), which will help the traumatologist identify patients for whom FR can be defined. However, this is not without controversy—in many studies that attempt to define temporal cut points, a large percentage of the patients have associated traumatic brain injury (TBI), negatively influencing outcomes in this group (patients with combined TBI and extracranial injury) and confounding causes of death. Therefore, it would be useful to review the literature regarding the definition of futility in patients with severe TBI with and without extracranial trauma and trauma-induced coagulopathy (TIC), and the relation of the severity of TBI to the accuracy of established clinical laboratory and radiologic parameters, as well as blood transfusion cut points, in order to define futility in greater detail in the early stages of those patients’ care [1,2,3,4,7,9,10,11,12,13]. Approximately half of the patients involved in the studies leading to “futility cut points” also had TBI [7], and, therefore, those cut points could lead to erroneously early declarations of futility for patients suffering only non-cranial penetrative and/or polytrauma and erroneously late declarations of futility for patients suffering from combined massive TBI and non-cranial bleeding. The deconvolution of these effects is a logical and ethical challenge. By leveraging historical insights from futility based on TBI-only scenarios and superimposing those learnings on the evolving criteria for FR—such as the Suspension of Transfusions and Other Procedures (STOP) criteria—one can develop a roadmap based on clinical decision tools that can inform individual clinical situations for severely injured patients with combined TBI and non-cranial bleeding while allowing TBI-dominated bleeding without significant non-cranial injury to retain its 72 h window due to the mercurial nature of brain injury and recovery [7,14,15,16,17,18]. However, the adherence to various protocols (discussed below) and the reliance on specific parameters are quite variable [14,15,17,19,20]. In addition to a “call to arms” (i.e., more research to deconvolute these effects), this document is a suggestion as to “which arms to call” (i.e., when to leverage TBI learnings to assist traumatologists when making inevitable gestalt-based decisions in combined TBI and non-cranial bleeding) in order to make the best possible decision when faced with the ethical trade-off imposed by limited transfusion resources and the desire to know inevitable mortality before declaring futility. The declaration of futility should not be a single event during the course of resuscitation, but the periodic analysis of reliable bedside parameters with FTOs/TTOs would possibly allow for a more judicious use of blood products with patients not likely to survive resuscitation due to severe traumatic-induced hemorrhage [2,5,6,7,8,21].

## 2. The Attempt to Define Parameters which Predict Futility in Patients with Polytrauma and TBI: Refining the STOP Algorithm

The STOP criteria have defined a combination of parameters that a possess 100% positive predictive value (PPV) and a 100% specificity for predicting death in severely bleeding patients who require MT, which is crucial for avoiding false positives when assessing futility. These parameters, proposed to be collected at periodic FTOs/TTOs during the first four hours of trauma resuscitation in severely bleeding trauma patients, include severe hypotension, threshold lactate, traumatic cardiac arrest with a return of spontaneous circulation (ROSC), the Glasgow Coma Scale (GCS), and lysis at 30 min (LY30) on thromboelastography (TEG) in varying combinations and thresholds, such that the strict adherence to this protocol will hopefully prevent the misclassification of an otherwise moribund patient [2,6,7,8]. The proposed STOP criteria are reproduced in Table 1.

A major concern arises in declaring the cessation of traumatic resuscitation too prematurely based on arrival hypotension, elevated lactic acid, and increased LY30 in patients who would otherwise survive. Hypotension can be particularly confounded due to the lack of correlation between manual and monitored blood pressures which are often not reliable in patients with operative illnesses, particularly with the elderly [22,23]. The STOP criteria use field and arrival blood pressures as parameters for futility [7]. Automated blood pressure monitoring in the prehospital environment is quite common [24]. However, there is no mention as to whether this is a manual or an automated blood pressure reading which further confounds the use of hypotension as a marker of futility in their study [7]. There have been recent attempts which have been described to define specific transfusion cut points per hour which can reliably predict futility in patients receiving MT for severe trauma-induced hemorrhage. However, there has been a significant discrepancy noted regarding transfusion cut points as independent markers for futility which do not consider other parameters such as those from the STOP criteria [1,4,7]. For this reason, the STOP criteria do not include transfusion cut points or resuscitation intensity [7]. It is, therefore, useful to propose that the PPV and specificity of the STOP criteria could be validated more thoroughly if independent markers for the severity of TBI were included alongside transfusion cut points since, as the authors of the STOP criteria study noted, it is the TBI population that consumes the most blood products in their study [7]. This observation could explain the discrepancy in studies that have failed to determine transfusion cut points as independent markers of futility [1,2,3,4,5,6,7,8]. It is possible that those patients who had ptTBI or very severe hiTBI consumed far more products with fewer markers of hypoperfusion, just as in the STOP criteria study. Conversely, trauma patients without TBI would be much more likely to survive the proposed cut points of blood pressure, lactic acid, and LY30 compared to patients with TBI.

Other studies that have attempted to analyze transfusion thresholds or cut points to assist in delineating those least likely to survive MT, as well as other attempts to define futility in patients with hemorrhagic shock, have also not separated and accounted for the influence of severe TBI as a cause of death. This is an important fact because, despite more than three decades’ analyses of transfusion cut points as predictors of futility in severely bleeding trauma patients, it is only recently that highly predictive and specific parameters have been proposed to define early FR in patients with hemorrhagic shock [1,2,3,4,5,6,7]. In many of these studies, the cause of death may not have been hemorrhage but, instead, TBI. Therefore, it is difficult to discern the predictors of early death in these studies since a large percentage of the patients had an associated TBI, and the bedside parameters of the severity of TBI other than the GCS were not used to predict early mortality in these studies [1,3,4,8,25,26,27,28,29,30,31,32,33,34,35,36,37,38,39,40,41,42]. The failure of most of these transfusion cut point studies to separate TBI deaths from non-TBI deaths presents an opportunity to review the literature regarding the ability of bedside predictors of futility in patients with TBI to enhance the accuracy of the recent analysis of transfusion cut points for bleeding trauma patients with or without TBI [1,3,4,8,25,26,27,28,29,30,31,32,33,34,35,36,37,38,39,40,41,42].

The historical precedence for this confounding influence of TBI on mortality can be seen even in the Pragmatic, Randomized Optimal Platelet, and Plasma Ratios (PROPPR) trial, where a 1:1:1 fixed ratio of PRBC, plasma, and PLT was associated with reduced death due to hemorrhage at 24 h (secondary outcome) and greater hemostatic control at two hours (secondary outcome). However, this study did not show a primary endpoint improvement in overall all-cause mortality at 24 h [43]. One of the concerns with this landmark study, which justified the 1:1:1 ratio-driven resuscitation for MT, is that the significance of TBI-associated death was not considered. Likewise, the significance of TBI is not considered in the STOP trial or in any of the most recent papers concerning the definition of FR for patients receiving MT for catastrophic hemorrhagic shock associated with trauma [43,44,45]. Interestingly, the STOP criteria were based on the patient population of the PROPPR trial. Therefore, the confounding factor of death by TBI in a study that was devoted to evaluating the effect of 1:1:1 on hemorrhaging patients has carried over into a study that produced parameters that predict futility in severely hemorrhaging patients due to trauma. These severely bleeding TBI patients will have TIC as measured by laboratory and viscoelastic parameters [9,10,11]. Even the authors of the PROPPR trial were aware of the confounding influence of TBI on mortality. They stated that this will continue to be an issue in future trauma studies unless novel regulatory study design or technological solutions are developed [43].

In the STOP criteria study, the effect of TBI was noted as a marker of patients who would consume large amounts of blood products without commensurate signs of global shock [7]. We refer to these patients as exhibiting coagulopathy of traumatic brain injury (CTBI), which is heterogeneously defined in the literature [9,10,11]. Specifically, in the STOP criteria validation cohort, CTBI patients had hyperfibrinolysis marked by increased LY30 and significant metabolic derangements, and had a much higher mortality than the comparison cohort who did not have these abnormalities. It was noted that CTBI patients that presented with a TEG LY30 > 70% and hypofibrinogenemia were more likely to consume blood products and had a severe head injury with a head Abbreviated Injury Scale (AIS) score > 3. It was also noted that, although these patients had relatively low lactic acid (<12 mmol/L) and, therefore, less hypoperfusion, the presence of CTBI resulted in a greater use of blood products. This would give impetus to the STOP criteria to include markers of the severity of TBI in an attempt to further refine the definition of futility in patients with combined TBI and severe extracranial trauma causing hemorrhagic shock. The severe TBI patients who had less severe shock than the comparator group demonstrated the classic findings of CTBI including early platelet dysfunction, hypofibrinogenemia, and the consumption of coagulation factors (which is a result of the uncoupling of the cardiovascular and autonomic nervous systems), as well as endothelial dysfunction and maladaptive hyperfibrinolysis [7,9,10,11]. The authors of the STOP criteria study saw the TBI population as a fertile source of reduction in blood product use during FR, stating:

*“The authors feel that this is the population in which we can expeditiously apply our findings to and provide an opportunity for resource stewardship. Locally, these patients have been described by several of our faculty as ones we are able to rescue on the macro level (with ROSC and often making it the OR), but fail to capture them on the micro level (with endothelial, coagulation, and electrophysiological uncoupling).”*

[7]

This review will focus on the same group of TBI patients that the authors of the STOP criteria identified, in whom futility could be defined earlier because of the concomitant presence of CTBI.

For the purposes of this review, we have delineated severe TBI into three categories. First, there is isolated TBI (iTBI). This is TBI with no extracranial traumatic injury and no concomitant coagulopathy and, thus, does not require blood products for resuscitation. The second type is hemorrhagic isolated TBI (hiTBI). This is TBI with no extracranial traumatic injury but with concomitant CTBI and necessitates MT. Finally, there is polytraumatic TBI (ptTBI). This is TBI with an extracranial traumatic injury and concomitant hemorrhagic shock and also requires MT. These polytraumatic injuries can be caused either by a penetrating or blunt injury to the brain and to the extracranial structures simultaneously. This classification of iTBI, hiTBI, and ptTBI could possibly assist clinicians in the future to predict FR more reliably in this large group of patients who consume significant quantities of blood components during resuscitation. It is necessary to delineate these three types of severe TBI because of the historical lack of reliable bedside predictors of futility in these populations [14,15,17,37,46,47,48,49,50,51,52,53].

## 3. Historical Definitions of Severe TBI in Relation to Transfusion Cut Points and Early Declaration of Futility

Before iTBI, hiTBI, and ptTBI can be separately defined, a review of the convoluted history of defining the severity of TBI based on the traditional mild, moderate, and severe parameters that have formed the staple of many definitions of TBI must be considered [14,15,16,17,19,20,25,51,54,55,56]. The definition of the grades of severity for TBI remains an object of controversy since the prognosis of iTBI varies from institution to institution, and the predictors of excellent recovery have not been confirmed in the literature, unlike prognostic indicators of futility for patients with a burn injury where prognostic indicators have evolved since the early 1960s [57]. Standard definitions of the severity of TBI have been focused on parameters such as age, pupillary reactivity, GCS without sedation, the presence of shock, traumatic cardiac arrest with ROSC, hypotension, and hypothermia [25,51,54,55]. Emblematic of the difficulty in arriving at a definition of futility is the adherence to a 72 h waiting guideline prior to determining futility and the use of variable parameters that are heterogeneous and institution-dependent [14,15,16,17,19,20,56].

There is considerable variability in individual aspects for the complex classification of the severity of TBI that have been based on nonspecific and non-standardized decision rules and classifications. These definitions and predictors of severity, mortality, and long-term disability have not yielded reproducible results. The use of computerized axial tomography (CT) scanning has allowed for a more dynamic definition of severity and prediction of mortality. TBI has been historically defined as mild, moderate, and severe based on the GCS, whereby mild has a GCS of 13–15, moderate has a GCS of 9–12, and severe has a GCS of 3–8 [14,15,16,17,51].

The prognosis is a function of the primary injury, which is grouped into contusions of the brain, epidural hematoma, subdural hematoma, intraparenchymal hemorrhage, intraventricular hemorrhage, subarachnoid hemorrhage, and diffuse axonal injury. Secondary injury consists of those changes subsequent to the initial insult and is characterized by system hypotension, hypoxia, hematoma expansion, and increased intracranial pressure (ICP). Clinical and radiologic indicators of severity include cerebral edema with or without a midline shift and brain herniation, which is divided into uncal, central transtentorial, cerebral tonsillar, falcine, and upward posterior fossa/cerebellar herniation. Each of these categories of herniation is associated with classical pupillary, motor, and pathologic reflexes, which allow for an early clinical identification, which can then be confirmed early in the stages of resuscitation with imaging [58,59,60,61,62].

Because of the lack of consistent parameters for predicting futility, new approaches toward the prediction of futility can be based on a multimodal, quantifiable platform. This includes imaging and biomarkers for futility, which would allow for the development of so-called risk labels, which are more predictive and specific than those markers of severity based solely on GCS [51,63,64].

Currently, many tools for assessing the severity of TBI are based on the GCS. However, the GCS is not a reliable measure of severity since it can be assessed at various times after the injury with different outcomes. Additionally, sedation and intubation during transport further obfuscate the interpretation of the GCS [65,66]. Furthermore, in the TBI patient with severe extracranial hemorrhage and shock, a depressed GCS can confound the estimation of TBI severity due to the fact that brain dysfunction may reflect the shock state resulting in brain hypoperfusion rather than severe TBI (i.e., a low head AIS score with low GCS) [67].

Other more reliable predictors of death following iTBI, such as the Corticosteroid Randomization after Significant Head Injury (CRASH) model and the International Mission for Prognosis and Clinical Trials in Traumatic Brain Injury (IMPACT) calculator, rely on variables such as age, secondary injuries, comorbidities, and brain imaging findings [46,50,51,53,68]. The seminal CRASH model and IMPACT calculator are designed to predict mortality at 14 days and 6 months, respectively; yet, they remain the foundation upon which subsequent predictors of severity and mortality are based [69]. The combination of different predictors with different time frames for defining mortality has allowed for the development of assessment tools that may provide an improved prediction for patients with severe TBI. Guidelines that include more information have been proposed, although there has been difficulty in delineating between moderate and severe TBI in predicting prognosis [68]. The heterogeneity of scores has resulted in an environment where the prediction of mortality in patients with iTBI leaves the traumatologist in a situation where competing algorithms default to the standard 72 h waiting period for neuroprognostication with a low level of adherence to guidelines for withdrawing care [14,15,16,17,46,50,53,56,70].

The 72 h limit before the declaration of death is subject to individual interpretation; studies have shown that patients can achieve independence after the 72 h period. It is for this reason that the declaration of futility should be considered for those patients who have such severe traumatic brain injury associated with an extracranial injury with known predictors of futility that have both a specificity and PPV of 100%. Due to the heterogeneity of the pathophysiology of TBI, whether penetrating or blunt, it can be difficult in patients without an extracranial injury to declare futility, even after 72 h, because of the occasional improvement in those patients with severe isolated TBI. However, it is reasonable, ethical, and safe to propose that patients with severe TBI associated with an extracranial injury who are in severe hemorrhagic shock due to bleeding and TIC are unlikely to survive 72 h. In addition, even though patients with an isolated blunt or penetrating TBI present with different pathophysiologies, the presence of other markers of futility, such as severe coagulopathy, advanced age, BFDP, and shock, further indicate that these patients will not survive.

However, there is considerable variance in the application of decision-making tools in withdrawal of care (WOC) decisions in the situation of what is called catastrophic brain injury or devastating brain injury (DBI). The variance is so great that it has been recommended that “neuroprognostication be based on individualized assessment of risk factors rather than on clinical scoring tools” [15,58,61]. However, given the potential for neurologic recovery, it has been noted that physicians and families engaging in shared decision-making often resort to dramatic lifesaving interventions with the realization that some of these patients will recover to a state of so-called “undesired life”. The adherence to these 72 h waiting time guidelines prior to declaring futility suggests that it is critical to assess for other comorbidities and pathologic processes that may mask an eventual prognosis. For patients with associated polytrauma and hemorrhagic shock, clearly, these additional pathologic markers enhance the predictability of death. [14,15]. It is of interest that, in the IMPACT trial, patients with certain death were described to have a GCS of 3 with fixed and dilated pupils [46,47,52,63]. Yet, even these two criteria have been found not to have a 100% PPV and specificity in order to effectively define futility [47,58].

The parameter of age is considered particularly significant in the foundation studies that define the severity of TBI, although the effect of the increasing severity of frailty on the outcomes is more important and can be added to the list of predictors of mortality for the geriatric trauma patient, such as the Geriatric Trauma Outcome Score (GTOS) and the Brain Injury Guidelines (BIG), which require the calculation of a post-admission Injury Severity Score (ISS) [71,72,73,74,75,76,77,78,79,80,81]. In the most recent studies to identify predictors of FR in patients with TBI, the accurate scoring of a “frailty index” in trauma patients can prevent unnecessary interventions in those with a high risk of dying while not withholding intervention in those who may find it beneficial [79,80]. However, the frailty index requires the calculation of parameters that cannot be calculated at the bedside within the first few hours of arrival; therefore, there is an impetus to find triage tools for patients with TBI that can predict early mortality [32,74,76,79,82,83,84,85,86,87,88].

For patients with severe extracranial injuries with hemorrhagic shock who require MT and for whom there have been recent algorithms to predict futility, such as the STOP criteria, it would seem logical and useful to incorporate bedside markers for the severity of TBI as independent predictors of FR. Few studies (such as the STOP criteria study) have proposed cut points to define futility in patients who have received MT for hemorrhagic shock and have evaluated various clinical parameters with and without TBI separately [1,7,14,15,17,37,46,47,48,49,50,51,52,53]. Even the STOP criteria include the GCS as its lone predictor of brain injury, and GCS used alone has been shown to be an insensitive and unreliable predictor of mortality in patients with TBI [7,51,65,66,69,89,90,91].

The goal would be to enhance pre-existing algorithms such as the STOP criteria so that futility could be more accurately predicted by adding the bedside parameters of the severity of TBI as independent risk factors with the addition of transfusion cut points per hour added to the parameters in those patients with ptTBI who are undergoing MT for hemorrhagic shock.

## 4. Defining iTBI, hiTBI, and ptTBI in Relation to Early Declaration of Futility

**Figure 1:**
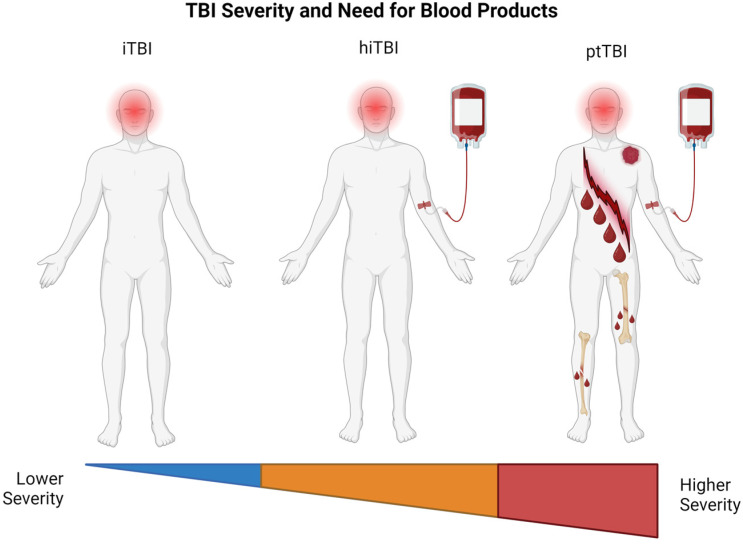
Types of severe traumatic brain injury arranged in ascending severity and blood product need from left to right. iTBI is TBI with no extracranial traumatic injury and no concomitant coagulopathy and, thus, does not require blood products for resuscitation. hiTBI is TBI with no extracranial traumatic injury but with concomitant CTBI and necessitates MT. ptTBI is TBI with extracranial traumatic injury and concomitant hemorrhagic shock and also requires MT. These polytraumatic injuries can be caused either by penetrating or blunt injury to the brain and to the extracranial structures simultaneously. Created using BioRender.com. hiTBI, hemorrhagic isolated traumatic brain injury; iTBI, isolated traumatic brain injury; ptTBI, polytraumatic traumatic brain injury; TBI, traumatic brain injury.

Table 2 and Figure 1 define the three categories of patients with TBI with their periods of observation before determining futility based on the literature and relation to coagulopathy [92,93].

For this discussion, emphasis will be placed on those patients with iTBI, hiTBI, and ptTBI who are candidates for the early cessation of FR. Patients in the hiTBI and ptTBI groups will have coagulopathy as measured by common coagulation tests and/or viscoelastic testing [9,11]. Currently, patients with hiTBI and iTBI traditionally have required long periods (up to 72 h) of evaluation before determining futility and, therefore, will not be candidates for the early determination of futility [14,15,16,17]. However, patients with hiTBI often die before the 72 h period, yet consume large amounts of blood products [3,7,93], and, as mentioned, we aim to leverage these findings to improve the declaration of futility in massive hemorrhaging. Guidelines for the WOC of patients with hiTBI are based on those for patients with iTBI without hemorrhage who are expected to survive 24 h. There is, however, a concession that patients with hiTBI with severe coagulopathy will survive less than 72 h [15,61,93]. Yet, since many of these patients will die before they can become organ donors, it would be beneficial to apply the standards of futility from the STOP criteria and add them to the nascent literature, which describes an early death in patients with severe iTBI, coagulopathy, and hypotension due to uncontrolled hemorrhage [1,4,7,58,61]. This uncontrolled hemorrhage with hiTBI, by definition, will not only occur within the brain but also in gastrointestinal, musculoskeletal, visceral, retroperitoneal, intrathoracic, and genitourinary areas affected by the overwhelming consumptive coagulopathy often associated with severe TBI [3,9,11,93].

The definition of iTBI is quite heterogeneous in the literature, but the most commonly accepted definitions are determined by an AIS head score ≥ 3 and an AIS extracranial score < 3. This defines a head injury in the absence of any other significant injury that would contribute to a coagulation disorder. The development of coagulopathy will predispose the patient with iTBI to require blood component therapy (BCT), and these patients have a worse prognosis [9,11,93].

It is important to note that the criteria for the AIS head score ≥ 3 is not universal [9,10,11]. Such heterogeneity has resulted in difficulty in determining the severity of TBI. Another approach is to define the severity of TBI based on the severity of coagulopathy associated with TBI, which has been shown to predict mortality. However, this definition of coagulopathy is not universal. For the purposes of this review, the most commonly used definition of iTBI is those patients with an AIS head score ≥ 3 and an AIS extracranial score < 3 [9,10,11,92].

In this group of patients with iTBI and AIS head scores ≥ 3 and AIS extracranial scores < 3 who are severely bleeding, they also may have acidosis and hypotension in the absence of polytrauma, which, therefore, contributes to the poor prognosis. We refer to these patients in our paper as those with hiTBI. These patients have a worse prognosis than those with iTBI without hemorrhage because of the presence of severe coagulopathy. Yet, these hiTBI patients are often resuscitated with blood components to treat the coagulopathy in the hope that they will survive long enough to become organ donors [58,61,93,95]. CTBI is often associated with disseminated intravascular coagulation (DIC). TBI patients with DIC have a much worse prognosis than those without DIC [3,9,11,93]. Specifically, the literature has recently described the increased mortality of patients with hiTBI, which can be attributed to the unique pathophysiologic characteristic of the release of tissue factor into the general circulation and its effect on systemic hemostasis [3,9,11,91,92]. The unique distinction between the coagulopathy caused by TBI and the coagulopathy caused by extracranial injury is a matter of ongoing research and controversy [12,13,93].

Patients with iTBI without hemorrhage or coagulopathy form one end of the spectrum of TBI patients who do not consume blood products, and they traditionally must wait 72 h before the declaration of futility, although this has been shown to be a standard that is inconsistently followed [14,15,16,17]. At the other end of the spectrum (i.e., ptTBI), those patients who require large quantities of blood components are those who form the bulk of the patient population and receive MT for hemorrhagic shock and for whom bedside algorithms for futility would be designed. In between these two poles of TBI severity are those patients with hiTBI who, because of a severe underlying coagulopathy related to their isolated head injury, will not survive for long periods and for whom the early declaration of futility will also need to be defined.

## 5. General Definition of Futility in TBI

Before comments can be made about a separate category of severe TBI for patients with ptTBI, a discussion regarding previous historical parameters and the timing of the definition of futility in patients with iTBI is important since the parameters and the timing of WOC in patients with ptTBI depend on this previous history for declaring futility in patients with iTBI. As mentioned above, the universally accepted standard for the period during which patients with iTBI need to be monitored for futility is 72 h prior to declaring brain death [14,15,16,17,18]. For example, a patient with severe TBI with fixed and dilated pupils, a GCS of 3, and tonsillar herniation accompanying severe extracranial injury with hemorrhagic shock will have a much worse prognosis than one without extracranial injury. However, the iTBI patient without an extracranial injury may have to wait 72 h before futility is declared. The ptTBI patient with an extracranial injury and hemorrhagic shock requiring MT may not survive the 72 h traditionally required for patients with iTBI prior to the consideration of a declaration of futility. However, the severity of TBI in the severely bleeding ptTBI patient adds an increased appreciation of futility during continued resuscitation, and, therefore, the addition of separate parameters or markers of futility of TBI in that polytrauma setting may assist in any identification of futility. [1,2,3,4,5,6,7,8,96,97,98]. An intervention is considered medically futile when it is unlikely to result in the meaningful survival for the patient [96]. The purpose of withdrawing life-sustaining treatment when care is futile is two-fold. First, continuing aggressive treatment for these patients can prolong suffering and cause more harm than good. According to the Society of Critical Care Medicine Ethics Committee, intensive treatment is inappropriate when there is no realistic expectation that the patient will survive or that the patient’s neurological functionality will return to a level high enough to allow for the perception of the benefits of the interventions [97,98].

Second, FR can result in the wastage of important resources that could be better allocated [1,2,3,4,5,6,7,8,98]. In the setting of TBI, certain resources may be limited, such as blood products, medicine, electroencephalogram machines, hospital beds, and provider time spent at the bedside [98]. However, withholding resources without evidence of futility remains controversial. Neurocritical care patients possess prognostic uncertainty, creating difficulty in transfusion-related futility decisions [98]. Approaches to transfusion limitation in the oncological, non-transplant, and non-survivable trauma populations have been reported. A similar concern for the wastage of blood components for patients in the neurocritical care unit with hiTBI remains an area in need of further research [93,98].

The literature regarding the determination of futility in patients with hemorrhagic shock with and without TBI predicts early mortality within 1 h, 6 h, and 24 h, and late mortality at 28 or more days [1,2,3,4,5,6,7,8,78,80,81,82,99]. Markers for death in patients with TBI that attempt to predict early death within 6 h or 24 h would be useful parameters to evaluate for the analysis of the patients with TBI and hemorrhagic shock. For the purposes of this discussion, where an attempt is made to delineate parameters in patients with TBI who will die, it is best to look at early markers in the literature and to divide the analysis of the timing of predicting FR into the prehospital, ED, and post-ED periods.

### 5.1. Defining Futility in iTBI

To date, there is variation in the literature regarding the ability to predict early death in patients with TBI. Recently, there has been an attempt to apply a quantitative algorithm to withdrawing care for patients with TBI. Whenever this decision is made, the risks and benefits of delaying prognostication regarding futility vary between institutions and patients, and recent research hopes to improve the accuracy of prediction tools which will reduce the risk of inaccurate prognostication [100].

The current literature mostly relies on studies that have attempted to define FR in TBI patients after admission to the hospital. These studies use the endpoints of death and/or disability at discharge rather than early death [32,74,76,79,80,82,83,84,85,86,87,88]. However, few studies evaluate parameters that anticipate FR in the prehospital and ED settings where resuscitation efforts may be terminated [32,54,55,83,85,101,102,103,104]. An often-cited reason for this reluctance is the desire to avoid a self-fulfilling prophecy (SFP), which has been cited as a negative aspect of the early declaration of FR in TBI [56]. In addition, it is still unknown if there are reliable parameters that allow for the accurate prediction of FR in TBI patients in prehospital and ED settings. There are, however, parameters and guidelines for severely hemorrhaging patients that allow for early WOC without TBI, which are the STOP criteria [7,56].

There are specific markers that can be used to define futility based on their ability to predict death at discharge and after. Specifically, markers such as age, mechanism, ISS, cardiac arrest, blood pressure, pupillary reactivity, GCS, length of stay in the ICU, the presence of penetrating injury, pre-existing morbidities, loss of gray-white matter on CT scan, brainstem hemorrhage, presence or absence of cerebral hemorrhage, fever, herniation, and prior use of anticoagulation have all been used in an attempt to define mortality at discharge within the first six months [58,60,69,77,78,79,80,81,105]. These studies offer a baseline from which to establish parameters that can be used to define mortality in the early hours of resuscitation in the prehospital, ED, surgical, and ICU treatment phases.

Given that there is literature that attempts to identify specific parameters in patients with iTBI who will survive discharge, there is some literature that has identified potential parameters for defining early WOC. Specifically, the timing of WOC in patients with severe TBI that have alterations in brainstem reflexes has been noted to be variable, and there has been a concern for a methodological type of SFP in using these brainstem reflexes as a marker for the declaration of early futility in this group of patients [106,107]. Neurologic function, divided into spontaneous activity, responsive activity, and communicative activity, has been used to define early mortality [100]. In addition, structural markers such as skull fractures, facial fractures, epidural hematomas, subarachnoid hemorrhage, cerebral contusion, and cerebral edema with and without a midline shift and/or cerebral herniation have all been shown to offer a certain degree of accuracy in defining early death in adults [58,59,60,61,100].

TBI is an important comorbidity, whether isolated or associated with hemorrhagic shock. Predicting futility is difficult for patients with TBI in the ED. An example of this difficulty in predicting certain death is that, even with an associated traumatic cardiac arrest after TBI, survival has been demonstrated among patients who recovered with good neurologic function [81]. However, the presence of coagulopathy in a patient with TBI who is consuming blood products is a very simple and clear-cut indication of a poor prognosis. Therefore, a discussion regarding a specific definition of FR in patients with hiTBI who are in hemorrhagic shock is a useful category for independently identifying early declarations of futility so as to conserve blood products while not denying these patients the opportunity to survive.

### 5.2. Defining Futility in hiTBI

Patients with iTBI traditionally require 72 h before a declaration of futility. Patients with hiTBI may also require 72 h in order to determine futility, although many will not survive this due to advancing coagulopathy [69,93]. Both iTBI and hiTBI patients are often sources of organ donation, and, therefore, the use of BCT with continued resuscitation is considered appropriate [17,19,20,58,61]. However, patients with hiTBI will often have severe coagulopathy, and, to continue providing blood components for 72 h, a clear understanding of the pathophysiologic contribution of TBI to the underlying CTBI is important not only for resuscitation but also for the declaration of futility [93]. A significant quantity of blood components will be used in this group of patients, as seen in the STOP validation cohort [7], and this can be anticipated in those situations where the patients are not able to live long enough to provide an organ donation.

For example, an evaluation of the odds ratio of CTBI and mortality with the parameters of age, large volume of venous fluids, GCS < 8, ISS > 16, AIS score = 5, subarachnoid hemorrhage on CT, cerebral edema on CT, midline shift on CT, abnormal pupils, hypotension, anemia, hyperglycemia, base deficit > 6 mmol/L, and shock index (SI) > 1 has been shown to affect the outcome in patients with TBI and also affect the likelihood that they will have coagulopathy [9,11,58,61]. The realization of the effect of these types of parameters on the presence of coagulopathy and the prognosis may assist the traumatologist in guiding the goal-directed BCT in these patients with hiTBI. Information regarding clinical factors that contribute to poor prognosis and mortality can assist the traumatologist in attempting to prolong the lives of donor transplant candidates and providing futility guidance for patients who have hiTBI.

It is difficult to separate patients with iTBI and hiTBI based on clinical and laboratory markers of coagulopathy since there are many different criteria for what constitutes coagulopathy in a patient with TBI [9,11]. However, a patient with severe TBI in the absence of extracranial trauma who is bleeding from every orifice and intravenous site because of DIC clearly has a worse prognosis than a patient with iTBI with no hemorrhage [3,9,93].

Therefore, for the patient with hiTBI, the duration of life could be measured in hours because of continued bleeding due to DIC, and these patients may be candidates for the early cessation of resuscitation should they require significant quantities of BCT in the first 2–6 h. There is little literature concerning this group of patients, a group that is not an insignificant percentage of patients who require MT in a busy metropolitan hospital [98].

### 5.3. Timing of Prediction of Futility in iTBI and hiTBI: Prehospital, ER, OR, and ICU

The early determination of futility occurs in three settings: prehospital, ED, and OR/ICU. Our purpose is to define reliable parameters for detecting FR in TBI patients in these three settings with or without hemorrhage. A recent study using a machine-learning protocol that relied on a GCS of 3–4, fixed pupils, and age concluded that it is difficult to use prehospital markers to define FR for TBI [106]. Confirming the difficulty in predicting certain death in patients with TBI, either in the prehospital or ED environment, is the fact that even the most recent guidelines from neurocritical care societies have stated that WOC for patients with TBI must wait 72 h after admission [14,15,17,52,69,107,108,109].

### 5.4. Defining Futility in ptTBI

As mentioned before, the STOP algorithm allows for the termination of resuscitation for patients with shock caused by traumatic hemorrhage with or without TBI. There has been no distinction in this study regarding the negative impact of TBI on this group of patients since patients who participated in the STOP criteria study were not stratified separately for severity at the bedside based on whether they presented with TBI or not other than by the GCS [7]. However, when using various BCT per hour cut points for determining FR, there has been controversy regarding the arbitrary selection of a number of units per hour to determine futility [1,2,3,4,5,6,7].

This recent controversy in the literature regarding the definition of parameters that allow for the ethical- and evidence-based termination of resuscitation of bleeding trauma patients with or without TBI supports the need to review the acceptable parameters for the withdrawal of resuscitation in hemorrhage due to ptTBI. The presence of TBI as a specific additional marker for patients who have an associated extracranial penetrating or blunt trauma may further refine the parameters and hourly transfusion cut points to define futility and allow for the early cessation of resuscitation when indicated [1,2,3,4,5,6,7,8].

### 5.5. Timing of Prediction of Futility in ptTBI: Prehospital, ER, OR, and ICU

Since bedside prediction tools for futility for severely bleeding ptTBI patients are in their earliest states, the prediction of FR during the withdrawal of resuscitation efforts is now being considered more by traumatologists caring for patients with traumatic hemorrhage due to the existence of these nascent predictors of early death and because of the looming shortage of the blood supply [1,2,3,4,5,6,7,14,15]. As noted above, the timing for the prediction of death for patients with TBI has traditionally been extended to 72 h. However, because of the recent push for early definitions of futility in order to prevent blood product wastage, resuscitation periods have been measured for those patients with severe ptTBI in the range of 2–6 h. These bedside prediction tools, which now allow for the early definition of futility within that 2–6 h period, have evolved from older parameters that were not able to be collected within the first hours of hospitalization [1,2,3,4,5,6,7,8,94]. A review of the history of the development of these bedside prediction tools must consider the foundation of these prediction tools and the evolution from this foundation upon which these bedside prediction tools are based.

## 6. The Evolution of Bedside Futility Markers for Hemorrhaging Patients with TBI and Trauma

### 6.1. From Clinical Gestalt to Metric-Assisted Gestalt: Addressing Equitable Distribution of Scarce Blood Products during MT

The STOP criteria were derived and validated from the PROPPR trial which established the criteria for predicting MT in trauma patients [7,43]. These criteria for predicting MT evolved from the variable thresholds for initiating MT, which were often a function of a so-called “clinical gestalt”. Clinical gestalt is the theory that clinicians actively organize clinical perceptions into coherent wholes based on previous exposure and experience which allows for better pattern recognition, particularly in times where decisions have to be made quickly [110,111,112]. In the hectic moments of resuscitation of the dying trauma patient, the decision to terminate resuscitation is often formed from the gestalt based on the clinical judgments of the physician’s past experiences, which are unique and subject to bias. However, it has been noted that the gestalt-driven decision for the determination of MT did not truly predict with accuracy the instances where MT would be needed [1,2,4,5,6,7,8,33,113,114,115,116]. This inaccuracy in gestalt-driven decisions regarding FR has driven the search for bedside, clinical, and laboratory parameters that will predict certain death in bleeding trauma patients with a 100% PPV and specificity [1,3,4,7,31,117,118,119].

In an effort to address the equitable distribution of scarce blood products to severely bleeding patients, traumatologists often face an ethical decision based on clinical observations and experience when making a judgment call in a time-sensitive environment during the administration of large quantities of blood products when efforts may appear futile, potentially limiting therapeutic resources for other patients [110,115,120]. These decisions to terminate resuscitation could be facilitated by the use of evidence-based studies demonstrating reliable markers for defining FR for exsanguinating trauma patients. However, the literature has not yet provided enough guidance, but there is nascent evidence that certain parameters and transfusion cut points may allow for a “metric-assisted” gestalt decision-making process for defining FR for the severely bleeding trauma patient. The most appropriate method of applying metric-assisted gestalt decision-making would be through the use of serial FTOs/TTOs during the early stages of resuscitation [1,2,3,4,5,6,7,8,115].

### 6.2. Non-Bedside Predictors of Late Mortality for TBI Patients

Guidelines such as the ISS, AIS, and Acute Physiology and Chronic Health Evaluation (APACHE) scoring systems are commonly employed to predict the prognosis for trauma patients in prehospital, ED, trauma centers, operating rooms, hospitalization, post-discharge, and intensive care unit (ICU) settings [79,80,121,122]. However, these are not bedside tests.

Few studies that prognosticate mortality at or after discharge rely on bedside markers [122]. Future research needs to explore the various parameters for determining FR in all types of TBI and analyze the pathophysiologic contribution of TBI to what we would think would be a significant bedside marker to enhance the accuracy of algorithms for FR.

As we have seen, the definitions of TBI and severe TBI have a history that is based on the GCS, pupillary reactivity, and the presence of an extracranial injury as described by the CRASH and IMPACT scores [46,53]. Subsequent attempts to identify patients who will die of TBI have led to algorithms that define mortality well after the 24 h mark and are based not only on the CRASH and IMPACT criteria but also on algorithms such as the ISS which cannot be calculated in the first hours of resuscitation for hemorrhagic shock. Since age has been shown to be a fairly reliable marker for early mortality, many studies have recently attempted to further quantify the effect of age and associated comorbidities in order to predict death after discharge. Examples of these scores are the GTOS, BIG score, frailty score, and GERtality score [71,72,73,74,75,76,77,78,79,81,82,84,86,99,123]. However, these above-mentioned protocols, which predict futility well after admission, may form the foundation for the development of bedside predictors.

### 6.3. Prehospital and Early Bedside Predictors of Early Mortality in Patients with TBI

While there have been recent advances in predicting 24 h and post-discharge mortality among patients with TBI, there have been nascent attempts to define mortality in the prehospital and early hospital periods for patients with TBI. For example, the Japanese Association for Acute Medicine (JAAM) Focused Outcomes Research in Emergency Care in Acute respiratory distress syndrome, Sepsis, and Trauma (FORECAST) study group has demonstrated that iTBI and high JAAM DIC scores in patients immediately after arrival to the hospital were highly predictive of in-hospital mortality [93]. An example of a bedside calculable algorithm for geriatric trauma is the Elderly Mortality After Trauma (EMAT) score, which is calculated upon admission to describe the probability of in-hospital mortality [32]. Such prehospital algorithms for elderly patients with TBI could assist in properly triaging these patients to palliative care more quickly as the prediction models become more robust with further study. The EMAT calculator can be considered an incipient tool to be used in the future in an attempt to provide machine-learning clinical scores that could assist in the prediction of futility in the prehospital and early environments in resuscitating moribund TBI patients [32]. Additional tools include the Pre-hospital Injury Mortality score [72,102], Supergeriatric score [77], Shock Index (SI) [85], prehospital ABC score [83], and the Commands, Age, Pulse rate, Systolic blood pressure, and O_2_ saturation (CAPSO) model [101,106].

With this foundation of recent publications that have attempted the early definition of mortality and that have been based on clinical studies that have been able to confirm the reliability of predictors of mortality for TBI patients at discharge, the trauma community now has a foundation with which to propose bedside markers for patients with TBI and hemorrhagic shock, which may refine the ability of the STOP criteria to identify FR in these ptTBI patients.

## 7. Proposal to Combine STOP Criteria with the Most Recently Accepted Guidelines for Early WOC of TBI: Adding Palliative Timeout to FTO/TTO

The STOP criteria have used markers of shock, such as the degree of fibrinolysis and serum lactate, to predict FR in trauma patients [7]. At selected periods during resuscitation, the use of FTOs has been proposed [3,7,8,119].

The markers for the STOP criteria were derived from the same markers used to initiate MT in the PROPPR trial. It has been shown that markers that predict the need for MT underestimate those patients who actually require MT in the setting of acute hemorrhagic shock due to trauma and are inaccurate [113,114,115,116]. However, the accuracy of these markers to predict FR must be 100% since withholding more transfusions for an anticipated FR will result in certain patient death. Hence, the threshold for determining FR requires a much higher degree of precision and accuracy. The goal of the STOP criteria study was to produce markers with a 100% PPV so that these markers would only identify those patients who truly would die regardless of the continued administration of blood products during resuscitation. The STOP criteria also required a 100% specificity so that no patients who would survive with continued hemorrhagic resuscitation would be falsely considered a candidate for WOC. These two strict statistical benchmarks, as clearly delineated in the STOP criteria, also need to be met for determining FR in TBI patients. A future analysis of the effect of severe TBI on massively bleeding trauma patients should focus on the further refinement of the criteria for FR [1,4,7,115,119,124].

The authors of the STOP criteria study have noted that nearly half of their patient population had TBI; yet, there is no distinction for those terminally bleeding trauma patients who also had an associated TBI. The study found that a GCS of 3 was a predictor of futility in adult trauma patients [7]. However, for patients in severe hemorrhagic shock without TBI, a GCS of 3 is also a marker of FR, and, therefore, the GCS as a marker of futility for TBI patients is confounded by this factor. We propose the combination of the STOP criteria with commonly cited markers of mortality for patients with TBI. The expansion of the STOP criteria to include independent TBI criteria for mortality would refine the accuracy of determining FR in hiTBI and ptTBI patients.

An example of novel methods that could be added to the markers for hiTBI and ptTBI is the “Double Diamond of Death” thromboelastography (DDD-TEG) or equivalent tracings in other viscoelastic tests that demonstrate extreme hyperfibrinolysis and certain death in trauma patients receiving MT [117,118,119].

ICP and cerebral perfusion pressure (CPP) have not been shown to be clinically useful tools to guide the treatment of individual TBI patients. However, these parameters are recognized as valid predictors of mortality. There is a clear association between the time spent above specific ICP and CPP thresholds and long-term prognosis [21,69]. Prediction models in TBI, which are usually static, rely on admission parameters and have a wide range of accuracy in predicting mortality [50,125]. However, a machine-learning tool that uses data based on ICP, mean arterial pressure (MAP), and CPP called the *ICP-MAP-CPP Algorithm* measures dynamic alterations of these three parameters over time and has been shown to predict mortality within 120 h of admission [21]. While the accuracy of this algorithm depends on the dynamic approach of analyzing multiple features derived from ICP, CPP, and MAP data over time, this study validates the utility of these three parameters in predicting FR in TBI patients. Thus, ICP and CPP may be candidates for inclusion in the STOP criteria for hiTBI and ptTBI.

Likewise, a new trauma score optimized for prehospital patients with TBI called the NTS-TBI was pioneered and has shown promise in predicting mortality and disability in the prehospital setting using motor GCS, hypotension, and hypoxia instead of the GCS, SBP, and respiratory rate of the revised trauma score (RTS) [126,127]. The discriminative power of this NTS-TBI score was significantly higher than that of the RTS, which includes some variables that are not easily obtainable in the field. This takes advantage of the fact that the NTS-TBI parameters can be acquired by paramedics in the field prior to any intervention. A similar prehospital tool for predicting mortality in the prehospital environment called the Early Warning Score (EWS) has led to the development of the National Early Warning Score tool (NEWS) which can help EMS personnel differentiate TBI patients with a high risk of deterioration and conserve another parameter to assist in the prediction of FR in patients with ptTBI [128]. Conceivably, a similar dynamic analysis within the first 6 h would allow for a more accurate definition of FR in TBI patients.

Another method that may be added is portable automated infrared pupillometry (PAIP), which allows for the automated and highly reproducible evaluation of both pupillary size and reactivity. PAIP represents an advancement in the ability to predict certain death in patients with severe TBI who are also in hemorrhagic shock (i.e., ptTBI) and, therefore, are candidates for declaring FR [129]. The addition of the PAIP to the arsenal of neuroprognostication could be considered in the same light as the addition of the DDD-TEG and the dynamic ICP-MAP-CPP measurements within the early hours of trauma resuscitation, whereby, when combined with other early markers of futility in TBI, it could enhance the accuracy of determining futility in patients with ptTBI and hiTBI [7,21,118,119,129]. Machine-learning protocols are being developed in order to assist traumatologists in the more accurate triage of patients in the early periods of trauma resuscitation [21,130]. The recent attempts to predict mortality through the prehospital and early assessment of the severity of injured elderly trauma patients could form the foundation for machine-learning protocols [32,72,83,101,102,103,126,128,130]. Future studies considering novel biomarkers when added to standard predictors of mortality have been proposed and offer the hope that a more granular and reproducible set of parameters will be developed in the future which will allow for the prehospital and early ED/OR/ICU determination of futility for TBI patients [131,132]. Table 1 contains the proposed STOP criteria, which have a 100% specificity and PPV for determining futility [7]. There are also neurocritical care guidelines as well as prehospital and hospital bedside parameters which have been described above that have demonstrated the ability to predict early mortality in patients with severe TBI with varying accuracy. In the future, these parameters will, hopefully, allow for the independent estimation of futility when added to the validated criteria (such as the STOP criteria) for futility in severely bleeding trauma patients who require MT.

Although the STOP criteria have demonstrated a 100% PPV and specificity as predictors of early death, the literature is lacking when it comes to the employment of serial FTOs/TTOs, or, similarly, a palliative timeout (PTO), as it concerns futility in the resuscitation of trauma patients receiving MT. [1,4,7]. However, in the literature concerning FR for patients with TBI, ethical considerations regarding the WOC are the standard, albeit with a significant variation in the application of varying protocols for declaring futility in patients with TBI [14,18]. Therefore, in addition to the FTOs/TTOs proposed by those who would limit resuscitation efforts based on clinical scores and transfusion cut points, a consideration of a PTO could be implemented during the FTOs/TTOs. An example of such a process has been proposed for patients with penetrating DBI [15,61]. In addition, this protocol addresses the allocation of scarce resources and allows for the incorporation of end-of-life care guidelines that respect the patients and their families [61].

Early Guiding Care Principles for Brain Injury, adapted from Zakrison et al. [61]:

1. Limit early and potentially premature neuroprognostication prior to hemodynamic stabilization;
2. Optimize individual patient outcomes;
3. Engage in a reasonable allocation of scarce resources;
4. Incorporate patient-respecting, end-of-life practices including organ donation as part of the continuum of compassionate care to patient and family

**Figure 2:**
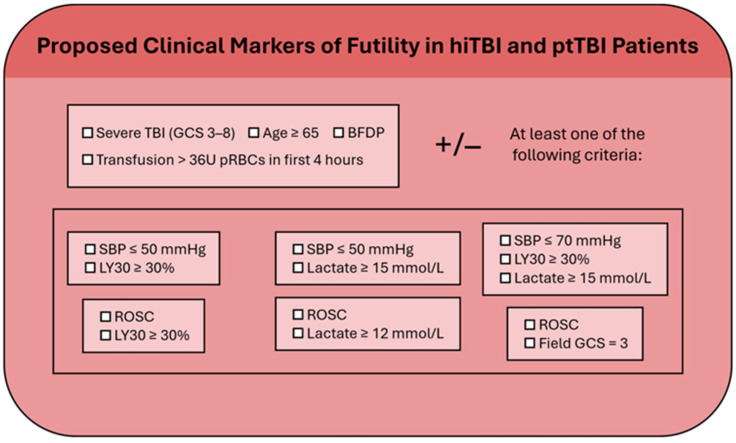
hiTBI and ptTBI patients undergoing massive transfusion for hemorrhagic shock. Potential markers during FTOs and TTOs for further research [1,3,4,7,58,119]. Adapted with permission from Ref. [7]. 2023, Van Gent (GCS and BFDP without paralysis or sedation).

In summary, the authors of the STOP criteria study noted that patients with TBI who had fewer markers for hypoperfusion, but a greater consumption of blood products were a group where these criteria would be most effective in reducing blood product consumption. The presence of coagulopathy, which is more frequently associated with patients with ptTBI, has been noted by the authors of the STOP criteria study as a significant risk factor for mortality, for which the authors used the term “coagulopathy equals death”. MT during the resuscitation of patients at risk for FR is a manifestation of TIC which is exacerbated by the coagulopathy associated with TBI. The STOP criteria do not consider this important predictor of CTBI and death by combining patients with and without TBI in their study and validation groups. The authors of the STOP criteria study have great faith in their parameters to predict certain death. However, initial blood pressure readings in the prehospital location and emergency department and the decision of which of a number of variable blood pressure readings, either manual or automatic, should be used when making this important decision are an example of how blood pressure is a capricious clinical parameter in the setting of acute trauma [22,23,24]. Therefore, it would be useful to have more reproducible and concrete parameters of certain death in severely bleeding trauma patients upon which to base the important decision to withdraw care. The GCS combined with BFDP as a marker for the severity of TBI is a very straightforward and reliable predictor of death, although there are outliers in the patients with iTBI that have rendered this a problematic final parameter upon which to base death within 72 h [58,61]. However, if a field GCS of 3 with BFDP is combined with transfusion cut points designed to describe heroic or futile resuscitation by previous authors, these criteria of the severity of TBI and of the coagulopathy of trauma associated with such massive quantities of blood would allow for a more refined and physiologic prognostic tool than those of the STOP criteria which must rely on blood pressure readings that are not always reproducible at low readings and on TEG LY30s that may not be available at all trauma centers [4,133]. In the STOP criteria study, there was no evaluation of transfusion cut points as potential markers for futility during the FTOs. Future studies could compare the STOP criteria alone with the STOP criteria plus markers for the severity of TBI and coagulopathy [7]. Future studies could be done to separate ptTBI from extracranial polytrauma. In these studies, one could hypothesize that the thresholds for withdrawing care as determined by the STOP criteria would be lower if the severity of TBI and the number of transfusion cut points were considered independent markers of futility to be added to the STOP criteria. For example, such a study could evaluate patients with a GCS between 3 and 8 when combined with transfusion cut points deemed heroic or at 16 units per four hours or futile at 36 units per four hours. The presence of BFDP would be an important addition as well. The addition of independent markers of very severe TBI and transfusion cut points could be used as part of a STOP criteria checklist to be adapted to the FTOs/TTOs (Figure 2). This hypothetical triage tool for assisting during FTOs/TTOs considers only the most extreme TBI with a GCS between 3 and 8, and BFDP, as well as the most severely bleeding patients with a cut point of 36 units PRBC in the first four hours. These criteria serve as a foundation from which to propose a graded scale whereby a GCS between 3 and 8 could be combined as well with the other markers from the STOP criteria study. In addition, markers such as age (as noted above) and DDD-TEG would also enhance the accuracy of such a proposed algorithm [1,3,4,7,58,61,119].

## 8. Ethical Implications of Defining Early TBI in the Presence of Shock

To date, there is little consensus regarding the widely accepted algorithms and guidelines that can predict FR with a high PPV and specificity for patients with TBI. Recently, however, there has been an increased recognition that the utilization of healthcare resources needs to be better focused on providing lifesaving care to those patients most likely to benefit [32,79,80]. In addition, significant inequities and disparities regarding the termination of FR in TBI have been noted. Race, location, and ability to pay are all correlated with WOC decisions, along with clinical parameters such as decreased GCS, increased age, and underlying conditions [18]. Not only is there a disparity in applying FR to patients with TBI based on the aforementioned factors, but there are also widespread inconsistencies in determining WOC in adults and pediatric patients with TBI. As a result, there has been an increase in attention paid to the inconsistency between the production of reliable parameters for FR in TBI, even in those settings where the prognosis is often bleak [14,15,17,32,58,61,134,135].

Low- to middle-income countries (LMICs) have confronted the problem of futility in patients with TBI with extensive epidemiologic analyses of their clinical, laboratory, and radiologic parameters that predict mortality [136,137]. In LMICs, the increase in TBI due to motor vehicle crashes and falls of the elderly is a consequence of a phenomenon called the ‘epidemiological transition’ whereby surgical-related illnesses, such as multiple trauma, overtakes infectious disease as the leading cause of death [138,139,140]. Therefore, the demand for medical services for these new trauma-related illnesses results in the increased utilization of and strain on healthcare resources [141]. This increased volume of surgical burden from noncommunicable diseases and trauma-related injuries has been correlated with increased life expectancy and the demographic changes that are a result of the increase in medical facilities, education, and enhanced mobility associated with growing socioeconomic development in the LMICs. The irony of this epidemiologic phenomenon of increased trauma related to improvements in medical and socioeconomic conditions has led many traumatologists to attempt to improve outcomes through prevention strategies and increased investment in high-level medical and surgical care. Yet, in the United States, a similar increase in the incidence of TBI due to falls and motor vehicle crashes has been noted, and, for those patients with trauma-induced hemorrhagic shock with TBI, the realization of a finite blood supply has caused the trauma community to search for reliable parameters to define futility [32,79,80]. Likewise, in the LMIC environment, there are similar attempts to identify FR more clearly in TBI so as to conserve scarce healthcare resources.

Similar to the literature in the United States regarding the definition of FR using transfusion cut points, the literature from LMICs regarding the definition of futility for patients who have suffered multiple trauma does not distinguish between those patients with TBI and those without TBI. This, therefore, bears a great similarity to the United States literature where the percentage of patients with severe injury due to multiple trauma associated with motor vehicle crashes also has a high incidence of TBI. Of interest is that, in high-income countries, there has been a recent proposal to limit blood products in the neurocritical care unit where futility is deemed likely for the same reasons that, in LMICs, early declarations of futility can likewise spare scarce resources so that others may benefit [83,98,123,136,142,143].

As was seen above, the declaration of futility in patients with iTBI in the absence of coagulopathy and polytrauma requires long periods of observation after admission into the critical care unit. Therefore, expanding on the previous discussion regarding the ethics of futility in patients with iTBI, we address the ethics of early WOC in the prehospital, ED, OR, and critical care units within 2–6 h for patients with severe TBI.

The ethical implications of denying patients care when there is a chance for survival have been a source of historical interest [90,107,144,145]. There are ethical implications in resuscitating a patient to a vegetative or nonoptimal state [144]. However, it has been noted that certain populations, such as the elderly, can be categorized as terminally ill, resulting in the so-called concept of SFP noted in the literature, which, when applied to TBI, occurs before 72 h [90,107,109]. The literature on the significance of SFP in trauma and non-traumatic deaths such as massive stroke and intracerebral hemorrhage can provide guidance so that the traumatologist may hopefully avoid the ethical fallacy of SFP in deciding FR for TBI patients. Further complicating the ethical discussion regarding the definition of FR for bleeding trauma patients is the significant variation in end-of-life care among trauma centers and the aforementioned low level of adherence to neurocritical guidelines for the definition of brain death, as well as the significant variation from institution to institution regarding the declaration of brain death for TBI patients [14,18,19,20,69,98].

There is a specific ethical consideration regarding the consumption of blood products: blood is considered to be living tissue, just like solid organs. Solid organ donation requires extensive eligibility criteria and an organized wait list for the purpose of rationing this scarce resource. In situations in which blood products are scarce, is it ethical to also use rationing strategies for the allocation of blood products [98]?

## 9. Conclusions

The recent use of whole blood and a 1:1:1 RBC, plasma, and PLT fixed ratio for severely bleeding trauma patients has placed significant strain on the blood supply [1,2,3,4,5,6,7,8,98]. In response, traumatologists have sought to define clinical, laboratory, and radiologic parameters that can accurately define those patients with severe traumatic hemorrhage who will not benefit from continued resuscitation in order to conserve blood component storage by blood banks. In addition, recent trends following the COVID-19 pandemic, with a reduction in donation sites because of remote employment, have also resulted in a clinical shortage of blood supply in the United States. In order to meet the rising demand for blood products for severely bleeding trauma patients while conserving existing blood storage, traumatologists have published guidelines such as the STOP criteria which have demonstrated a 100% specificity and 100% PPV for defining certain death among patients with severe traumatic hemorrhage. Nearly half of these patients in the STOP criteria study and other papers that have sought to define highly predictive parameters of certain death in the severely bleeding trauma population have confounding TBI. Yet, there was no attempt to add TBI markers of early mortality to the STOP criteria for patients who have associated TBI. Recent literature suggests the establishment of FTOs/TTOs during the hectic moments of hemostatic resuscitation for severely bleeding trauma patients [2,3,4,6,7,8]. The addition of independent markers that predict with a high degree of PPV and specificity a likely death in TBI patients would increase the accuracy of guidelines such as the STOP criteria and further reduce blood product wastage during resuscitation for severely bleeding trauma patients while preserving the highest possible level of predictive accuracy that is required when deciding to terminate resuscitation.

## Figures and Tables

**Table 1:** Proposed STOP criteria. Data from Van Gent et al. [7] Adapted with permission from Ref. [7]. 2023, Van Gent.

Variable	PPV, %	NPV, %	Sn, %	Sp, %
Arrival SBP ≤ 50 and LY30 ≥ 30%	100	78	33	100
Arrival SBP ≤ 50 and lactate ≥ 15	100	77	31	100
Arrival SBP ≤ 70, LY30 ≥ 30%, and lactate ≥ 15	100	77	30	100
ROSC and LY30 ≥ 30%	100	78	33	100
ROSC and lactate ≥ 12	100	76	29	100
ROSC and field GCS 3	100	77	27	100

Abbreviations: GCS, Glasgow Coma Scale; LY30, lysis at 30 min; NPV, negative predictive value; ROSC, return of spontaneous circulation; PPV, positive predictive value; SBP, systolic blood pressure; Sn, sensitivity; Sp, specificity.

**Table 2:** Abbreviations of TBI and common recommendation for period of observation before declaration of futility for patients receiving massive and ultramassive transfusion.

TBI Type	Hemorrhage and Coagulopathy	Abbreviated Head Injury Score	Abbreviated Extracranial Injury Score	Common Futility Wait Time Recommendations	Common Futility Wait Times in Clinical Practice
iTBI	No	≥3	<3	72 h	72 h
hiTBI	Yes	≥3	<3	Variable	<72 h *
ptTBI	Yes	≥3	>3	Variable	2–6 h **

* Traditionally 72 h for iTBI and variable for hiTBI, since severely coagulopathic patients with hiTBI may have a consumptive coagulopathy which precludes waiting 72 h in order to declare futility. ** Transfusion cut points per hour which define futility are determined during FTOs within the first 2–6 h [1,2,3,4,5,6,7,8,9,10,11,14,15,16,17,92,93,94]. Abbreviations: hiTBI, hemorrhagic isolated traumatic brain injury; iTBI, isolated traumatic brain injury; ptTBI, polytraumatic traumatic brain injury; TBI, traumatic brain injury.

## Abbreviations

ACP	American College of Physicians
AIS	Abbreviated Injury Scale
APACHE	Acute Physiology and Chronic Health Evaluation
BCT	Blood component therapy
BIG	Brain injury guideline
CCT	Common coagulation testing
ciTBI	Clinically important traumatic brain injury
CPP	Cerebral perfusion pressure
CRASH	Corticosteroid Randomization after Significant Head Injury
CT	Computerized axial tomography
CTBI	Coagulopathy of traumatic brain injury
DAI	Diffuse axonal injury
DBI	Devastating brain injury
DDD-TEG	Double diamond of death thromboelastography
DIC	Disseminated intravascular coagulation
EMAT	Elderly Mortality After Trauma
ETCO_2_	End-tidal CO_2_
FFP	Fresh frozen plasma
FR	Futile resuscitation
FTO	Futility timeout
GCS	Glasgow Coma Scale
GTOS	Geriatric Trauma Outcome Score
HIC	Higher-income country
hiTBI	Hemorrhagic isolated traumatic brain injury
ICP	Intracranial pressure
IMPACT	International Mission for Prognosis and Clinical Trials in Traumatic Brain Injury
ISS	Injury Severity Score
iTBI	Isolated traumatic brain injury
LMIC	Low to middle-income country
LY30	Lysis at 30 min
MAP	Mean arterial pressure
MT	Massive transfusion
PAIP	Portable automated infrared pupillometry
PLT	Platelets
PPV	Positive predictive value
PRBC	Packed red blood cells
PTO	Palliative timeout
ptTBI	Polytraumatic traumatic brain injury
ROSC	Return of spontaneous circulation
SAH	Subarachnoid hemorrhage
SFP	Self-fulfilling prophecy
SOFA	Sequential Organ Failure Assessment
STOP	Suspension of Transfusions and Other Procedures
TBI	Traumatic brain injury
TEG	Thromboelastography
TF	Tissue factor
TIC	Trauma-induced coagulopathy
TRACULA	Tracts Constrained by Underlying Anatomy
VET	Viscoelastic testing
WOC	Withdrawal of care
